# Variation in Dube3a expression affects neurotransmission at the *Drosophila* neuromuscular junction

**DOI:** 10.1242/bio.20148045

**Published:** 2015-05-06

**Authors:** Colleen Valdez, Reese Scroggs, Rachel Chassen, Lawrence T. Reiter

**Affiliations:** 1 Department of Neurology, The University of Tennessee Health Science Center, 855 Monroe Ave., Link 415, Memphis, TN 38163, USA; 2 Department of Anatomy and Neurobiology, The University of Tennessee Health Science Center, 855 Monroe Ave., Link 515, Memphis, TN 38163, USA

**Keywords:** Axonal transmission, Spontaneous transmission, Dube3a, Ube3a, *Drosophila* neuromuscular junction, Synaptic transmission

## Abstract

Changes in *UBE3A* expression levels in neurons can cause neurogenetic disorders ranging from Angelman syndrome (AS) (decreased levels) to autism (increased levels). Here we investigated the effects on neuronal function of varying UBE3A levels using the *Drosophila* neuromuscular junction as a model for both of these neurogenetic disorders. Stimulations that evoked excitatory junction potentials (EJPs) at 1 Hz intermittently failed to evoke EJPs at 15 Hz in a significantly higher proportion of *Dube3a* over-expressors using the pan neuronal GAL4 driver *C155*-GAL4 (*C155*-GAL4>UAS-*Dube3a*) relative to controls (*C155*>+ alone). However, in the *Dube3a* over-expressing larval neurons with no failures, there was no difference in EJP amplitude at the beginning of the train, or the rate of decrease in EJP amplitude over the course of the train compared to controls. In the absence of tetrodotoxin (TTX), spontaneous EJPs were observed in significantly more *C155*-GAL4>UAS-*Dube3a* larva compared to controls. In the presence of TTX, spontaneous and evoked EJPs were completely blocked and mEJP amplitude and frequency did not differ among genotypes. These data suggest that over-expression of wild type Dube3a, but not a ubiquitination defective Dube3a-C/A protein, compromises the ability of motor neuron axons to support closely spaced trains of action potentials, while at the same time increasing excitability. EJPs evoked at 15 Hz in the absence of Dube3a (*Dube3a^15b^* homozygous mutant larvae) decayed more rapidly over the course of 30 stimulations compared to *w^1118^* controls, and *Dube3a^15b^* larval muscles had significantly more negative resting membrane potentials (RMP). However, these results could not be recapitulated using RNAi knockdown of Dube3a in muscle or neurons alone, suggesting more global developmental defects contribute to this phenotype. These data suggest that reduced UBE3A expression levels may cause global changes that affect RMP and neurotransmitter release from motorneurons at the neuromuscular junction. Similar affects of under- and over-expression of UBE3A on membrane potential and synaptic transmission may underlie the synaptic plasticity defects observed in both AS and autism.

## INTRODUCTION

Angelman syndrome (AS) is a devastating human neurological disorder characterized by cognitive and behavioral defects, muscle hypotonia as well as jerky limb movements and a debilitating ataxic gait (Williams, 2005). Mouse models of *UBE3A* maternal loss of function exhibit deficits in learning, hippocampal long term potentiation, and experience-dependent maturation of the neocortex (Jiang et al., 1998; Miura et al., 2002; van Woerden et al., 2007; Yashiro et al., 2009), which may represent alterations in calcium/calmodulin-dependent protein kinase II, properties of axonal initial segment, postsynaptic regulation of glutamatergic signaling, and dendrite morphogenesis (Haas et al., 2007; Kaphzan et al., 2011). The ataxic gait phenotype of AS is clearly recapitulated in mice deficient for *Ube3a* as demonstrated by rotarod performance, gait analysis, and cerebellar controlled licking behavior (Jiang et al., 1998; Miura et al., 2002; Heck et al., 2008). Although these gait phenotypes appear to be primarily due to a decrease in inhibitory signals in the cerebellum (Egawa et al., 2012), a comprehensive analysis of motor neuron function in the absence of UBE3A has not yet been performed and rescue of Ube3a levels in the cerebellum of *Ube3a* deficient mice does not always rescue the ataxic gait phenotype (Meng et al., 2013).

Duplications of the same region deleted in the majority of individuals with AS are the second most common genetic lesion (3-5% of cases) found in autism (Moreno-De-Luca et al., 2012). Just as maternal deletion is required for an AS phenotype, maternal duplications of 15q are specifically associated with increased autism risk (Cook et al., 1997; Urraca et al., 2013). A mouse model with a duplication syntenic to human interstitial duplications of 15q11.2-q13, displayed behavioral deficits characteristic of autism, possibly caused by a deficit in 5-HT2c receptor signaling (Nakatani et al., 2009; Tamada et al., 2010). These data support the hypothesis that the level of *UBE3A* expressed from the maternal allele in neurons is critical to neuronal development and function; deficiency for maternal *UBE3A* resulting in Angelman syndrome and duplication of maternal *UBE3A* driving increased autism risk.

*Drosophila* models of *Dube3a* deficiency [the orthologue to *UBE3A* in flies (Reiter et al., 2006)] have revealed that the loss of *Dube3a* in neurons results in decreased dendritic arborization in larval peripheral neurons (Lu et al., 2009), decreased dopamine levels in adult fly brain (Ferdousy et al., 2011), and a clearly measurable defect in climbing ability in adult flies (Wu et al., 2008). Wu et al. found that adult flies deficient for *Dube3a* or expressing wild type *Dube3a* in neurons showed significant defects in climbing ability that were ubiquitin ligase dependent, implying an underlying defect at the neuromuscular junction that may also depend on Dube3a ubiquitination (Wu et al., 2008). We previously showed that *Dube3a* loss of function causes changes in the expression of various protein components of the actin cytoskeleton eventually leading to a measurable loss of filamentous actin in the larval muscle wall (Jensen et al., 2013), so this effect may also be due to muscle developmental defects.

The fly neuromuscular junction (NMJ) is an excellent model for examination of genes involved in synapse formation, function and regulation (Bellen and Budnik, 2000), but can also be used to examine the effects post-synaptic defects in larval muscle on neurophysiology. Studies of mammalian synapses in the brain have pointed to a pivotal role for the ubiquitin proteasome system in both pre and post-synaptic regulation (DiAntonio and Hicke, 2004) and this is also true for the development and function of the fly NMJ (Haas et al., 2007). To find out how changes in Dube3a levels affected neuronal function (both axonal and synaptic) at the NMJ we examined synaptic transmission at 3^rd^ instar larval NMJ under conditions of both loss and over-expression of Dube3a. We identified defects in axonal propagation of action potentials and synaptic transmission associated with changes in Dube3a in motor neurons. This study provides evidence that the phenotypes observed in humans and mice with decreased or elevated *Ube3a* may be at least in part related to defects in axonal and synaptic function.

## RESULTS

### *Dube3a* Over-Expression

**Fig. 1.**
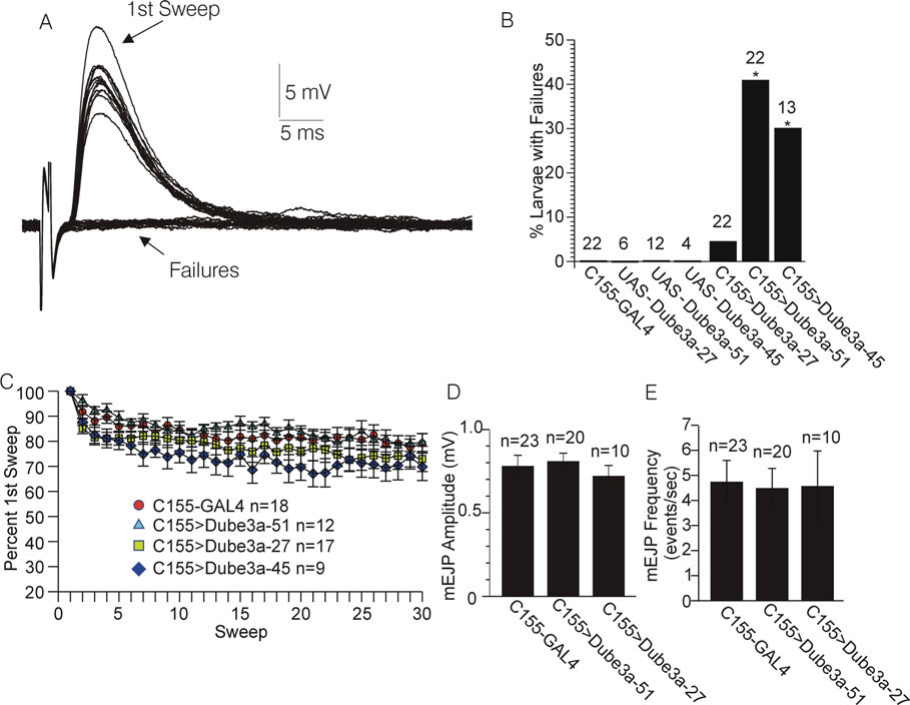
**Effects of over-expression of *Dube3a* on the change in evoked post synaptic potential (EPSP) amplitude during rapid (15 Hz) stimulation.** (A) Voltage record acquired from one *C155*>*Dube3a*-51 larvae expressing moderate levels of wild type Dube3a protein in neurons. Note the intermittent failure of the stimulation to evoke an EJP over the course of 30 stimulations at 15 Hz. (B) Percentage of larvae where failures were observed during the same stimulation protocol illustrated in A, for larvae over-expressing *Dube3a* at high levels (*C155*>*Dube3a*-27), or moderate levels (*C155*>*Dube3a*-51, *C155*>*Dube3a*-45) versus controls (*C155*>+ alone). The asterisk indicates a significantly higher frequency of larvae with failures compared to controls (p<0.05, Fisher's exact test, probability adjusted for multiple comparisons). Numbers above bars are the *n* value of larvae tested per genotype. (C) Plot of EJP amplitude versus time over the course of 30 stimulations at 15 Hz for larvae that did not exhibit failures in the same groups depicted in B. There were no significant differences between the groups shown (ANOVA, repeated measures, p>0.05). (D,E) mEJP amplitude (D) and frequency (E) for mEJPs recorded in the presence of the voltage-gated Na^+^ channel blocker TTX (3 μM) from *C155*>*Dube3a*-27, *C155*>*Dube3a*-51, and *C155*>+ larvae. There were no significant differences among the groups for either amplitude or frequency of mEJPs (ANOVA followed by Bonferroni test, *p*>0.05). Data are reported as mean±s.e.m.

A series of experiments were carried out to test the effects of over-expression of *Dube3a* on changes in evoked junction potential (EJP) amplitude at rapid stimulation rates. In 40-50% of *C155*>*Dube3a*-51 and *C155*>*Dube3a*-45 larva, stimulations at 15 Hz intermittently failed to evoke EJPs (Fig. 1A,B). For these experiments, a minimum stimulation voltage was used that was previously (∼1 min earlier) shown to consistently evoke EJPs at 1 Hz using the same nerve and at the same recording site. The proportion of *C155*>*Dube3a*-51 and *C155*>*Dube3a*-45 larvae where failures at 15 Hz were observed was significantly greater than failures observed in the *C155*>+ controls (Fisher's Exact test, p<0.05. Fig. 1B). No failures were found in UAS-*Dube3a*-51 (n=12), UAS-*Dube3a*-45 (n=4) or UAS-*Dube3a*-27 (n=6) animals in the absence of the GAL4 driver (Fig. 1B). Failures were found in only ∼10% of *C155*>*Dube3a*-27 larva, however, despite the fact that this allele expresses higher levels of Dube3a than UAS-*Dube3a*-51 and UAS-*Dube3a*-45 alleles (supplementary material Fig. S1). In the *C155*>*Dube3a*-51 (n=12) and *C155*>*Dube3a*-45 (n=9) larva where failures were not observed, the decrease in EJP amplitude over the course of 30 stimulations at 15 Hz did not differ significantly from that observed for the *C155*>*Dube3a*-27 larva (n=17) or the *C155*>+ controls (n=18) (ANOVA, repeated measures p>0.05, Fig. 1C). Interestingly, in 20% of *C155*>*Dube3a*-51 and 15% of *C155*>+ larva mentioned above, a small degree of short term facilitation (2-7% increase) was observed for the second EJP relative to the first EJP. There was no significant difference between *C155*>*Dube3a*-51, *C155*>*Dube3a*-27, or *C155*>+ larva regarding the amplitude of the first EJP in the train or the average resting membrane potential (RMP) over the course of the train (ANOVA, p<0.05, data not shown). Additionally, in experiments carried out in the presence of 3 μM TTX no significant difference was detected in mEJP amplitude or frequency between *C155*>*Dube3a*-45, *C155*>*Dube3a*-27, or *C155*>+ larva (ANOVA, p>0.05, Fig. 1D).

### Spontaneous EJPs

**Fig. 2.**
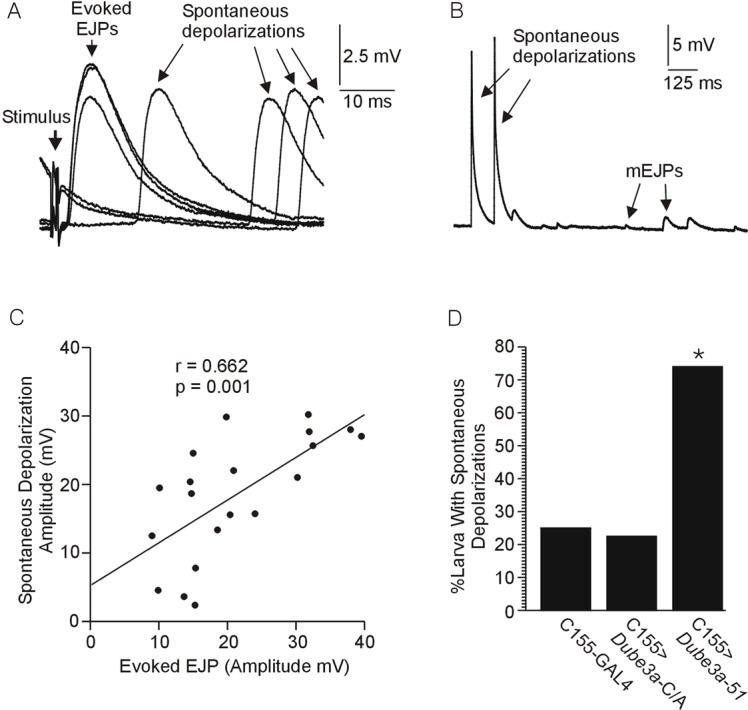
**Effects of over-expression of Dube3a on the occurrence of large spontaneous depolarizations.** (A,B) Large spontaneous depolarizations of the resting muscle membrane potential observed during recordings of EJPs (A) and mEJPs (B). The mEJP recordings were carried out in the absence of the Na^+^ channel blocker TTX, thus allowing for the propagation of spontaneous axonal discharges. (C) Plot of EJP amplitude (evoked) versus large depolarization amplitude (observed during mEJP recordings) in 20 cases where both measures were acquired during an individual recording at the same site. There was a significant correlation between the spontaneous and evoked amplitudes, p<0.05, Bartlett Chi-square. (D) Percentage of cases where large spontaneous depolarizations were observed during mEJP recordings in the absence of TTX from wild type Dube3a expressing (*C155*>*Dube3a*-51), ubiquitination defective (*C155*>*Dube3a*-C/A) and control larvae (*C155*>+). Note that the ubiquitin ligase function was required for this significant increase in the percentage of spontaneous depolarizations. The asterisk indicates a significantly higher proportion of larvae exhibiting spontaneous depolarizations in the *C155*>*Dube3a*-51 group (p<0.05, Fisher's exact test, probability adjusted for multiple comparisons).

During the above stimulation experiments, and also during additional experiments focused on mEJPs recorded in the absence of TTX, spontaneous depolarizations resembling EJPs in amplitude and duration were observed (Fig. 2A,B). There was a significant correlation between the amplitude of the spontaneous depolarizations observed during the recordings targeting mEJPs, and EJPs evoked by stimulation in the same larva (Fig. 2C). The spontaneous depolarizations were also observed in a significantly higher proportion of *C155*>*Dube3a*-51 larva relative to their *C155*>+ controls and relative to the expression of inactive Dube3a that can bind, but not ubiquitinate, substrates (*C155*>*Dube3a*-C/A) (Fisher's Exact test, Fig. 2D). Similar spontaneous depolarizations were never observed in mEJP recordings carried out in the presence of TTX. The spontaneous depolarizations usually occurred in bursts, often at fairly high frequencies around 15-20 Hz (Fig. 2B). Apparent short-term facilitation between the first and second spontaneous depolarizations was observed in about one-half of larva where spontaneous depolarizations occurred at high frequencies (Fig. 2B).

### *Dube3a* deficiency

**Fig. 3.**
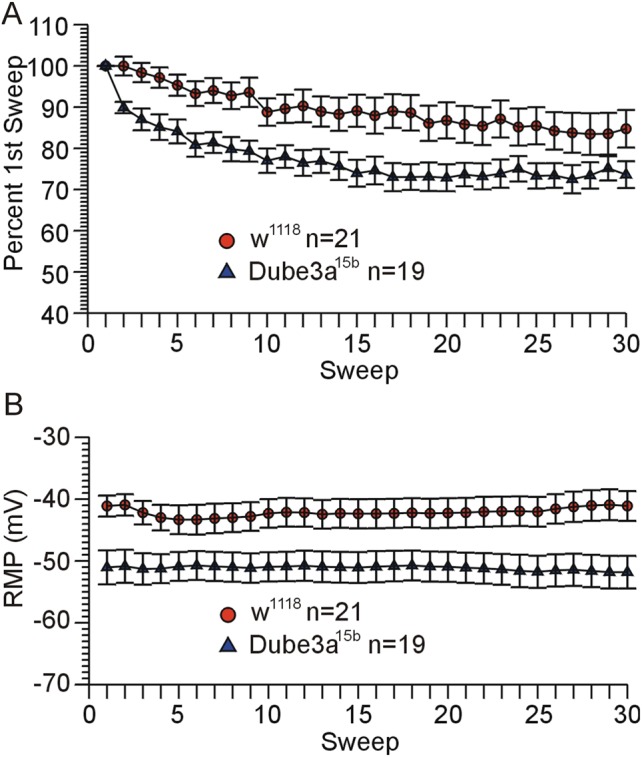
**Deficiency for Dube3a protein in *Dube3a^15b^* homozygous mutants causes a decrease in EJP amplitude and RMP during rapid stimulation.** (A) Graph of EJP amplitude versus stimulation number (Sweep) over the course of 30 stimulations at 15 Hz for *w^1118^;Dube3a^15b^/Dube3a^15b^* versus *w^1118^* larvae (significantly greater decrease for *Dube3a^15b^* (p<0.05, ANOVA, repeated measures). (B) RMP measured immediately before each stimulation for the same recordings depicted in A. The average RMP recorded over the course of each train of 30 stimulations was significantly more negative for *w^1118^;Dube3a^15b^/Dube3a^15b^* versus *w^1118^* larvae (p<0.05, Student's *t-*test). Data are reported as mean±s.e.m.

A series of experiments was carried out to test the effects of *Dube3a* deficiency on changes in EJP amplitude at rapid stimulation rates. EJPs recorded from homozygous *w^1118^;Dube3a^15b^* larva, which make no Dube3a protein (Wu et al., 2008), decreased in amplitude significantly more over the course of 30 stimulations delivered at a rate of 15 Hz than EJPs recorded from *w^1118^* controls (ANOVA, repeated measures, p<0.05, Fig. 3A). The average muscle RMP recorded over the course of each stimulus train was significantly more negative in in *Dube3a^15b^* larva compared to *w^1118^* controls (Student's *t*-test, p<0.05, Fig. 3B). No significant difference was detected between *Dube3a^15b^* larva and *w^1118^* control larva regarding the amplitude of the first EJP in the train (ANOVA with average RMP as a covariate, p>0.05). However, short-term facilitation was observed in significantly more *w^1118^* control larva (9 out of 21, range 3-34%) compared to *Dube3a^15b^* larva (1 out of 19, 2% increase, p<0.05, Fisher's exact test).

### *Dube3a* knock down in muscle and neurons

**Fig. 4.**
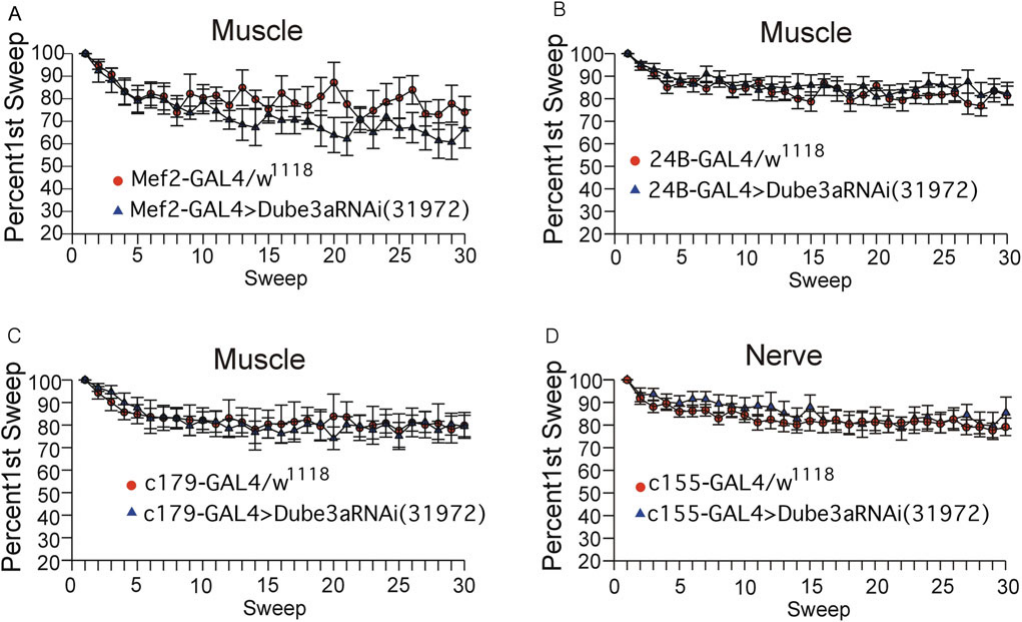
**Knockdown of Dube3a in either muscle or neurons alone does not recapitulate the *Dube3a^15b^* loss of function EJP and RMP phenotypes.** Graphs of EJP amplitude versus stimulation number (Sweep) over the course of 30 stimulations at 15 Hz for (A) *mef2*-GAL4>UAS-*Dube3a*RNAi(31972), (B) *24B*-GAL4>UAS-*Dube3a*RNAi(31972), (C) *c179*-GAL4> UAS-*Dube3a*RNAi(31972) and (D) *c155*-GAL4>UAS-*Dube3a*RNAi(31972). GAL4 drivers *mef2*, *24B* and *c179* all drive expression in muscle, while the *c155*-GAL4 driver is a pan neuronal expresser. At least n=7-11 control animals expressing the GAL4 alone in a *w^1118^* background were also analyzed for each driver as controls. Knock down of Dube3a in any of these muscle patterns or globally in neurons did not have an effect on either RMP or EJP, nor did it produce the spontaneous depolarizations observed for Dube3a overexpression as illustrated in Fig. 2. Data are reported as mean±s.e.m.

In order to separate muscle specific Dube3a loss from neuronal loss of Dube3a we attempted to reconstitute portions of the decreased EJP and RMP observed in *Dube3a^15b^* homozygous mutants using the GAL4/UAS system. To knock down Dube3a in larval muscle we used *c179*-GAL4 (which expresses in embryonic mesoderm, larval muscles and wing imaginal discs, n=9); *24B*-GAL4 (also known as *how*^24B^-GAL4 which expresses in both embryonic and larval somatic muscle, n=11); and *mef2*-GAL4 (which expresses in somatic muscle and imaginal wing disks, n=11). We found no significant differences in either RMP or EJP when we expressed a UAS dsRNAi against Dube3a in either muscle or neurons using the pan neuronal drive *c155*-GAL4, n=18 (Fig. 4). These results suggest that that despite a known muscle defect in Dube3a deficient larvae (Jensen et al., 2013) that knock down of Dube3a in muscle or neurons alone does not cause the defects in neuronal function observed in the homozygous loss of function mutants.

### Examination of physical changes to the synaptic active zone

**Fig. 5.**
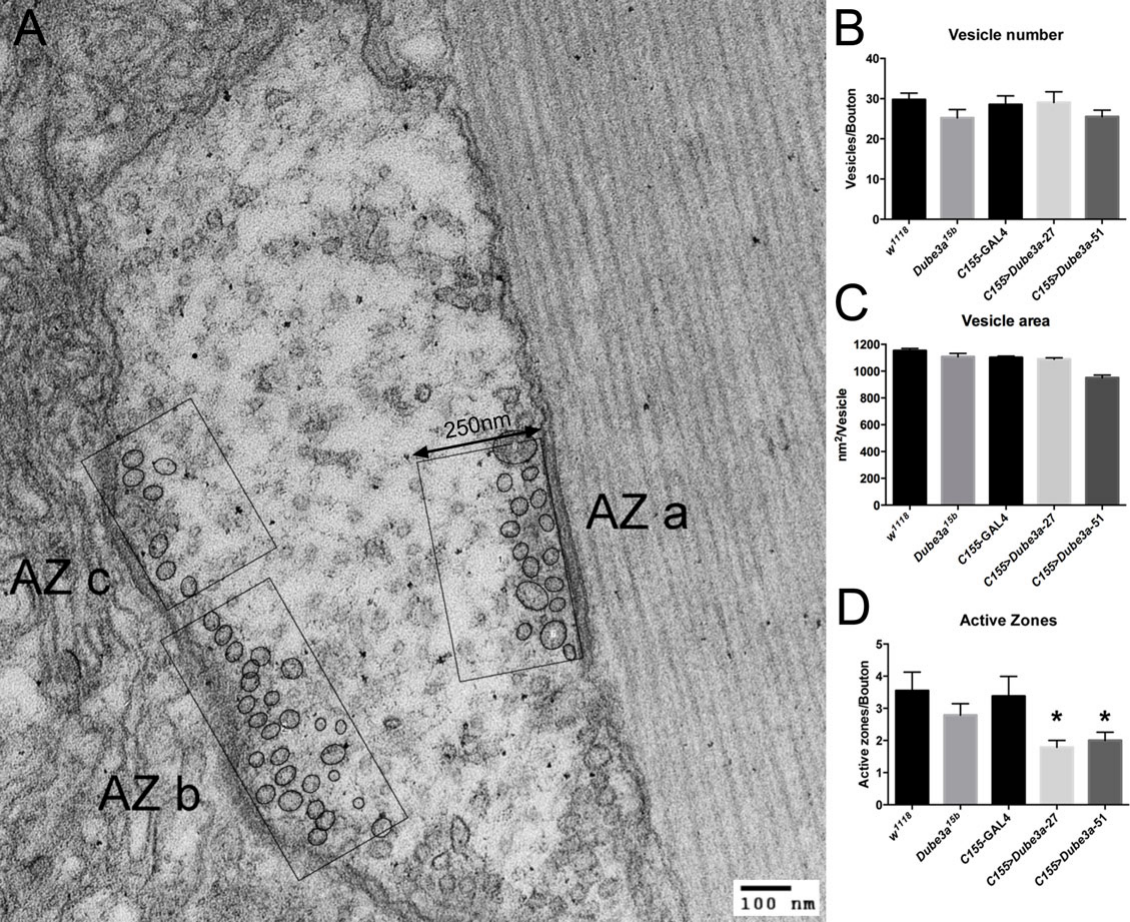
**Elevated Dube3a expression results in fewer active zones per bouton and a decrease in vesicle area.** (A) Representative image of transmission electron microscopy (TEM) analysis of 3rd instar larval synapses. The active zones were identified by an increase in density along the border of the bouton combined with the presence of synaptic vesicles. AZ stands for “active zone”. The image was taken at 60 kV and the scale bar indicates 100 nm. The region used for analysis on each active zone was 250 nm from the edge of the bouton in each case. (B) Vesicle number per active zone in each genotype was essentially unchanged. (C) The average vesicle area was measured for each genotype. Only animals expressing moderately high levels of Dube3a (*C155*>*Dube3a*-51) showed a significant decreased in average vesicle area. (D) Significant differences were found in the number of active zones detected per bouton. Larvae over-expressing either moderate (*Dube3a*-51) or strong (*Dube3a*-27) alleles of *Dube3a* had significantly fewer active zones per bouton than *C155*>+ larvae. The asterisks indicate significantly fewer active zones/bouton for these genotypes (p<0.05, Fisher's exact test, probability adjusted for multiple comparisons). Data are reported as mean±s.e.m.

Additional experiments examined the structure of synaptic boutons and active zones at the larval NMJ of animals over-expressing and deficient for Dube3a protein in neurons. Using two antibodies [the α–Nc-82 (bruchpilot) antibody to identify synaptic active zones and the Dlg (Discs-large) antibody to identify the larger synaptic boutons], we quantified both the number of boutons present on the muscle surface as well as the density of synaptic active zones within these boutons in four animals per genotype for all groups (supplementary material Fig. S2A,B). This analysis failed to reveal any significant differences in either the number of boutons or the density of active zones within these boutons for all genotypes (supplementary material Fig. S2B). The fine structure of the pre-synaptic active zones in all genotypes was also examined using transmission electron microscopy (TEM) in three animals per genotype and at least animals per genotype. The vesicle number per active zone was approximately equal among all genotypes (Fig. 5B). However, (assuming that each synaptic vesicle represented an independent data point) there were significantly smaller vesicles in *C155*>*Dube3a*-51 larva compared to all other genotypes (ANOVA followed by *t*-test corrected by Bonferroni adjustment for multiple comparisons, p<0.05, Fig. 5C). In addition, significantly fewer active zones were detected in both *Dube3a*-27 and *Dube3a*-51 over-expression larvae (ANOVA followed by Bartlett's correction, p>0.001, Fig. 5D). However, since active zone analysis requires the presence of at least one active zone to score a given bouton, these numbers could be artificially inflated.

## DISCUSSION

This study demonstrates that both over-expression and deficiency for *Dube3a*, the fly orthologue of human *UBE3A*, alters neurotransmission at the neuromuscular junction in *Drosophila melanogaster* 3^rd^ instar larvae. In a significant proportion of larvae expressing elevated levels of Dube3a in neurons, rapid stimulation of motor nerves intermittently failed to evoke an EJP, and spontaneous depolarizations resembling evoked EJPs were frequently observed in the absence of TTX. However, the amplitude of the first EJP in the train of evoked EJPs and the amplitude and frequency of mEJPs did not vary between any of the genotypes, indicating that this was an axonal rather than vesicle recycling issue. Also, in over-expressors that did not exhibit evoked EJP failure, EJP amplitude did not change more than controls during rapid stimulation. Finally, the spontaneous depolarizations were not observed in larvae over-expressing a ubiquitination defective form of Dube3a (Dube3a-C/A) indicating that the phenomena is dependent on the ubiquitin ligase function of the Dube3a protein (Fig. 2D). These data could be explained by assuming that evoked EJP failure and spontaneous depolarizations resulted from regulation of Dube3a ubiquitin target(s) in motor neuron axons rather than directly on the release of neurotransmitter at the synapse. One possible explanation for the spontaneous depolarizations and failures is that *Dube3a* over-expression results in a depolarization of the RMP of the motor neurons. It is possible that a depolarized membrane potential could result in inactivation of Na^+^ channels, which could lead to inability of the axons to conduct closely spaced action potentials. At the same time, depolarization of the membrane potential could increase excitability of the axon by bringing it closer to the potential where large numbers of Na^+^ channels begin to activate. Under this condition any minor perturbation of the axon membrane potential could trigger an action potential in the motor neuron and subsequent EJP in the targeted muscle. The spontaneous depolarizations often appeared as bursts, the termination of which might be also be explained by Na^+^ channel inactivation, similar to the intermittent failures observed at rapid stimulation rates. There was no significant difference in muscle RMP in *Dube3a* over-expressors versus controls, which is expected since the *C155*-GAL4 driver employed selectively targets neurons and not muscle.

Complete loss of *Dube3a* expression in the mutant resulted in a different pattern of effects from over-expression. In *w^1118^*; *Dube3a^15b^*/*Dube3a^15b^* larvae, which make no functional Dube3a protein, the EJP decreased more rapidly in response to rapid stimulation compared to their *w^1118^* controls (Fig. 3). This is typically referred to as short term depression (STD). The observation of apparent STD in *Dube3a^15b^* larvae could be related to the observation that short term facilitation (STF) was less frequently observed in *Dube3a^15b^* versus their *w^1118^* controls. STD is thought to be due to a depletion of the readily releasable pool of synaptic vesicles, while STF is thought to be the result of Ca^2+^ build up in the terminal due to rapid successive depolarizations (Desai-Shah and Cooper, 2009). At the stimulation rate of 15 Hz, the overall change in EJP amplitude could be a balance between STF and STD. Possibly, a deficit in STF in *Dube3a^15b^* larvae could have led to an overall faster decrease in EJP amplitude relative to *w^1118^* controls.

Also, the RMP in the muscles of *Dube3a^15b^* mutants was significantly more negative than their *w^1118^* controls. These data may reflect a deficit in one or more of the processes or elements involved in maintenance of the RMP. A recent study from our laboratory (Jensen et al., 2013) suggests that Na^+^/K^+^ ATPase is ubiquitinated in a Dube3a dependent manner. One might expect that if the loss of *Dube3a* is causing the more negative RMP in muscle via an effect on Na^+^/K^+^ ATPase levels or activity, then the motorneurons may also be affected because regulation of muscle and nerve cell membrane potential both depend on the Na^+^/K^+^ ATPase. We must acknowledge that changes in resting K^+^ levels due to leakage across the membrane could also explain our findings. However, it may be more than a coincidence that the effects of over-expression of *Dube3a* resulted in increased evoked EJP failures and increased spontaneous, both of which may be indications of a depolarized RMP in motor neurons in the corresponding larvae. Over-expression of *Dube3a* may have the opposite effects on RMP as loss of *Dube3a* via opposing actions on this ubiquitin target.

The data we present here on the structure of the synaptic active zones suggests that *C155*>*Dube3a*-27 and *C155*>*Dube3a*-51 larvae had fewer active zones and that *C155*>*Dube3a*-51 also had smaller synaptic vesicles relative to the other genotypes. We also show that there is a slight increase in synaptic zone density by NC82 staining (supplementary material Fig. S3B), however these results did not reach significance despite the large dataset analyzed. These effects of altered Dube3a expression do not seem to explain the effects of over-expression or deficiency on the electrophysiological paradigms employed. However, they may later prove to be important observations that explain deficits in synaptic transmission not tested in the present study. In mouse models of both Angelman syndrome (decreased Ube3a) and Duplication 15q autism (elevated Ube3a) there are defects in glutamatergic synaptic transmission (Yashiro et al., 2009; Smith et al., 2011). Here we have shown that these defects in glutamatergic signaling can be recapitulated in the fly models for both syndromes as well, validating the fly model system for both syndromes. Thus, in a simple and easy to manipulate model system, the *Drosophila* NMJ, we can now investigate the downstream effects of changes in Dube3a levels on potential ubiquitin targets in the context of neuronal function. Some putative Ube3a protein targets such as Arc and CamKII have been known for some time (Weeber et al., 2003; Greer et al., 2009), while an entirely new set of potential Dube3a targets was recently identified in our group through a proteomic screen in flies (Jensen et al., 2013). We anticipate that by manipulating the putative targets of Dube3a in the fly NMJ system through shRNAi knock down or mutations in these genes we can begin to unravel the molecular mechanism behind the neurological defects observed in humans with both AS and Duplication 15q autism.

## MATERIALS AND METHODS

### Fly (*Drosophila melanogaster*) stocks

The *w^1118^* stock, the pan neuronal GAL4 driver *C155*-GAL4 (an insertion into the fly *elav* locus) and three GAL4 larval muscle drivers (*24B*-GAL4, *c179*-GAL4 and *mef2*-GAL4) were obtained from the Bloomington Stocks Center (Bloomington, IN). The Dube3a dsRNAi stock is part of the Harvard TRiP UAS driven dsRNAi collection (Bloomington stock #31972). Stocks (*w^1118^*;UAS-*Dube3a*) and (*w^1118^*;UAS-*Dube3a*-C/A) have been published previously (Ferdousy et al., 2011). All UAS-*Dube3a* lines were generated from the same cDNA clone as described previously (Reiter et al., 2006). The UAS-*Dube3a*-27 line expresses the highest levels of Dube3a protein *in vivo* and shows a rough eye phenotype with the eye specific drive *gmr*-GAL4 while UAS-*Dube3a*-51 and UAS-*Dube3a*-45 both produce a moderate increase in Dube3a protein levels *in vivo* and show only a mildly rough eye with *gmr*-GAL4 (supplementary material Fig. S1A). Expression levels for transgenic Dube3a protein are also elevated in heads of *Heatshock*-GAL4>UAS-Dube3a animals in accordance with this rough eye phenotype (supplementary material Fig. S1B). All flies were raised at 25°C on standard corn meal media with the exception of the *C155*-GAL4; UAS-*Dube3a*-27 crosses which only produced viable larvae at room temperature (∼20°C).

### Larval dissections

Dissections were carried out in a chamber approximately 1 mm deep and 2 cm in diameter filled with a Drosophila saline solution containing (in mM): 128 NaCl, 1.8 CaCl_2_, 2 KCl, 5 MgCl_2_, 36 sucrose, 5 HEPES, adjusted to pH 7.2 with NaOH (Jan and Jan, 1976). Crawling 3^rd^ instar larvae were dissected under 4× magnification as previously described (Brent et al., 2009b). All of the segmental nerves that innervate the body wall muscles were cut near the brain. The preparation was washed with Drosophila saline several times to remove debris and any soluble agents released during the dissection. The average dissection time was under 5 min per larvae and no preparations were used past 30 min post dissection.

### Electrophysiology

All chemicals used in the experiments, including tetrodotoxin citrate, were purchased from Sigma-Aldrich (St. Louis, MO, USA). Sharp electrode current clamp recordings were carried out under static conditions in Drosophila saline and in the same chamber used for dissection. Electrodes ranging from 15-30 MΩ were pulled using a Flaming-Brown microelectrode puller model 97 (Sutter Instruments, Novato, CA, USA), and filled with 3 M KCl. With the aid of a dissecting microscope, recordings were initiated via impalement of larval muscle 6 in segment A3. The cut end of the 3^rd^ segmental nerve on the appropriate side was drawn into a suction electrode prior to recording the resting membrane potential and mini spontaneous excitatory junction potentials (mEJP). Data consisting of resting membrane potential (RMP) excitatory junction potentials (EJPs) using a Grass 220 stimulator were recorded from muscle and acquired using an Axoclamp 2B amplifier interfaced with a 1200 series Digitizer to a computer running Clampex 8.1 for analysis (Axon Instruments, Union City, CA, USA).

To study the effects of rapid stimulation rates on EJP amplitude and failure rate, a threshold voltage was first found which consistently elicited EJPs at a stimulation rate of 1 Hz. Then, following an approximately 1 min interlude, EJPs were again recorded at the same site and stimulation voltage, but at a stimulation rate of 15 Hz. Larvae were excluded from the analysis of rapid stimulation if the resting membrane potential varied more than ±5 mV. mEJPs were recorded in the same solution as EJPs, in the absence of tetrodotoxin (TTX) in some preparations, and in the presence of 3 μM TTX in other preparations. TTX was administered to the bath by infusing approximately 3 ml of Drosophila saline containing 3 μM TTX into the recording chamber via a syringe connected to the bath with small diameter tubing, while withdrawing solution from the other side of the bath at the same rate with a similar syringe-tubing setup. This procedure completely blocked EJPs elicited by nerve stimulation. mEJP amplitude and frequency was analyzed using the event detection program (template search mode) in Clampex 10.2 (Microdevices, Sunnyvale, CA, USA).

### Immunohistochemical staining of 3^rd^ instar larvae

Crawling 3^rd^ instar larvae of the appropriate genotypes were dissected in 4% paraformaldehyde fix solution as previously described (Brent et al., 2009a). Both the α-Nc-82 (1:500) and α-Dlg (1:500) monoclonal antibodies were obtained from the Developmental Studies Hybridoma Bank developed under the auspices of the NICHD and maintained by The University of Iowa, Department of Biology, Iowa City, IA, USA. Alexa Flour fluorescent secondary antibodies (α-mouse 488 and α-goat 594 both at 1:1000) were obtained from Life Sciences. Images were captured as a Z-stack through the entire bouton region on a Zeiss 710 confocal microscope available for use through the UTHSC Neuroscience Institute. Flattened images were then processed in ImageJ on the red (Nc-82) channel using the “find maxima” feature to quantify the number of synapses. Counts were repeated for at least 4 animals per genotype for both active zones and bouton number (Dlg). Statistics to determine if significant differences were present among genotypes were performed in Prism 5.0 software (GraphPad Software, San Diego, CA, USA).

### Electron microscope experiments

Crawling 3^rd^ instar larvae of the appropriate genotypes were dissected in gluteraldehyde fix solution (2% paraformaldehyde, 2% gluteraldehyde, 0.2 M of cacodylate and 0.005% CaCl_2_). Fixed larvae were then processed, embedded and sectioned (70 nm) by the UTHSC Neuroscience Institute Imaging Core. Transmission electron microscopy images were captured on a JEOL JEM-2000EXII microscope at 60 kV to visualize the active zones within NMJ boutons. Images containing boutons with clearly identifiable active zones were used for statistical analysis. A rectangular box extending 250 nm inward from the AZ and running the entire length of each AZ was used for analysis. Bouton volumes were measured using ImageJ. Statistical analysis via One-way ANOVA was performed using Prism 5.0 software (GraphPad Software, San Diego, CA, USA).

### Statistics

Data are reported as the mean±the standard error of the mean. The Student's *t*-test was used to test for differences between two independent groups, and analysis of variance, followed by a post hoc *t*-test (adjusted for multiple comparisons) was used to test for differences between 3 or more independent groups. Analysis of variance for repeated measures was used to test for differences between independent groups, where data consisted of repeated measures taken over time from individual larvae. The Fisher's exact test was used to test for differences between independent groups regarding the frequency of occurrence of an event. Probabilities less than 0.05 were considered significant.

## Supplementary Material

Supplementary Material
