# Supplementary material for: Variation in Dube3a expression affects neurotransmission at the Drosophila neuromuscular junction
Source: Biol Open. 2015 May 6;4(7):776–82. doi: 10.1242/bio.20148045 (PMC4571101; doi:10.1242/bio.20148045)
Supplement: Supplementary Material [file supp_4_7_776__index.html]

Variation in Dube3a expression affects neurotransmission at the Drosophila neuromuscular junction — Variation in Dube3a expression affects neurotransmission at the Drosophila neuromuscular junction — Supplementary Material 

# Variation in Dube3a expression affects neurotransmission at the *Drosophila* neuromuscular junction

## BIO20148045 Supplementary Material

- Supplementary Material
