# Supplementary material for: Variation in Dube3a expression affects neurotransmission at the Drosophila neuromuscular junction
Source: Biol Open. 2015 May 6;4(7):776–82. doi: 10.1242/bio.20148045 (PMC4571101; doi:10.1242/bio.20148045)
Supplement: Supplementary Material [file supp_bio.20148045_BIO20148045supp.pdf]

(A)

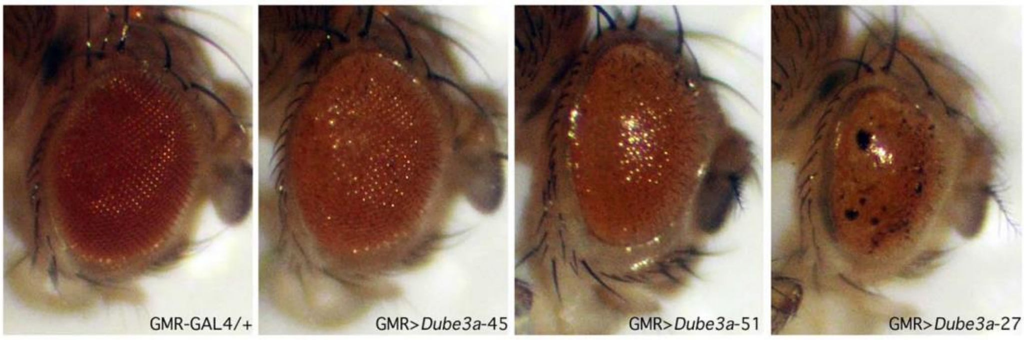

(B)

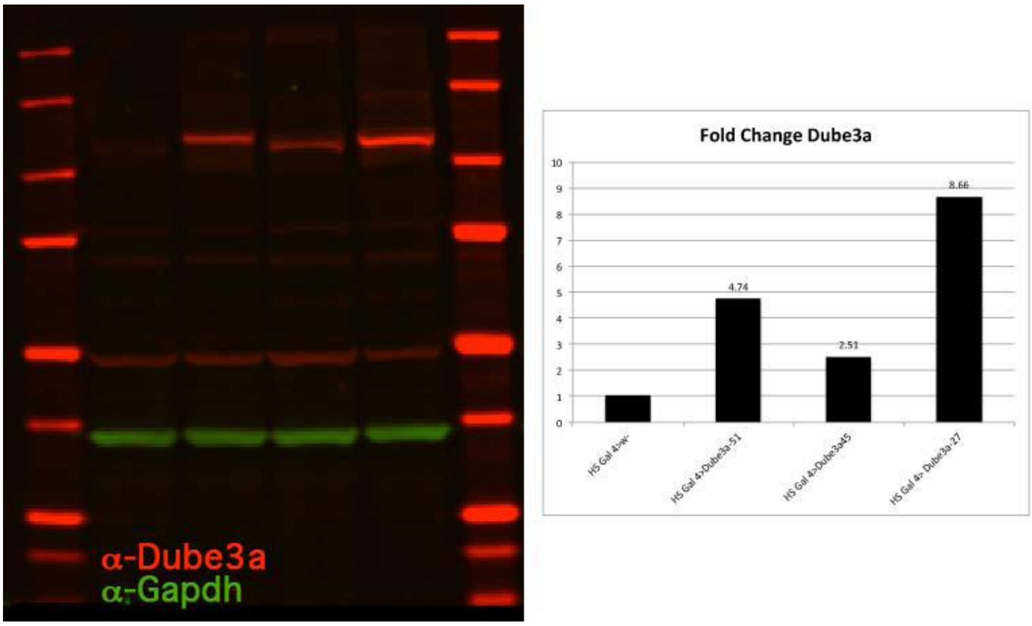

Fig. S1. Effects of different UAS-*Dube3a* insertions on expression of Dube3a *in vivo*. (A) All three UAS-*Dube3a* alleles were generated using the same cDNA clone (see Reiter et al., 2006 for details). The site of integration in the fly genome for each P-element is unknown, but it has a direct effect on the expression level for each transgene. Using the eye specific GAL4 driver (*gmr*-GAL4) we observed a rough eye phenotype for all three UAS-*Dube3a* transgenic lines. The weakest effect was seen with *Dube3a*-45 and the strongest with *Dube3a*-27, indicating that *Dube3a*-27 lines may express higher levels of the Dube3a transgene. (B) Quantitative western blot of Dube3a expression in heads expressing *Dube3a*-51, *Dube3a*-45 and *Dube3a*-27 as compared to *Heatshock*-GAL4 alone. Red band at ~120 kDa is Dube3a protein detected using α-Dube3a antibody provided by Dr. Janice Fischer (University of Texas, Austin, TX). Loading control is α-Gapdh. The right side panel is the fold changed in Dube3a protein intensity, normalized to Gapdh for loading and compared to the *Heatshock*-GAL4 control lane (lane 1). Expression of the transgenic Dube3a is analogous for each UAS allele to the roughness of the eye described in part A.

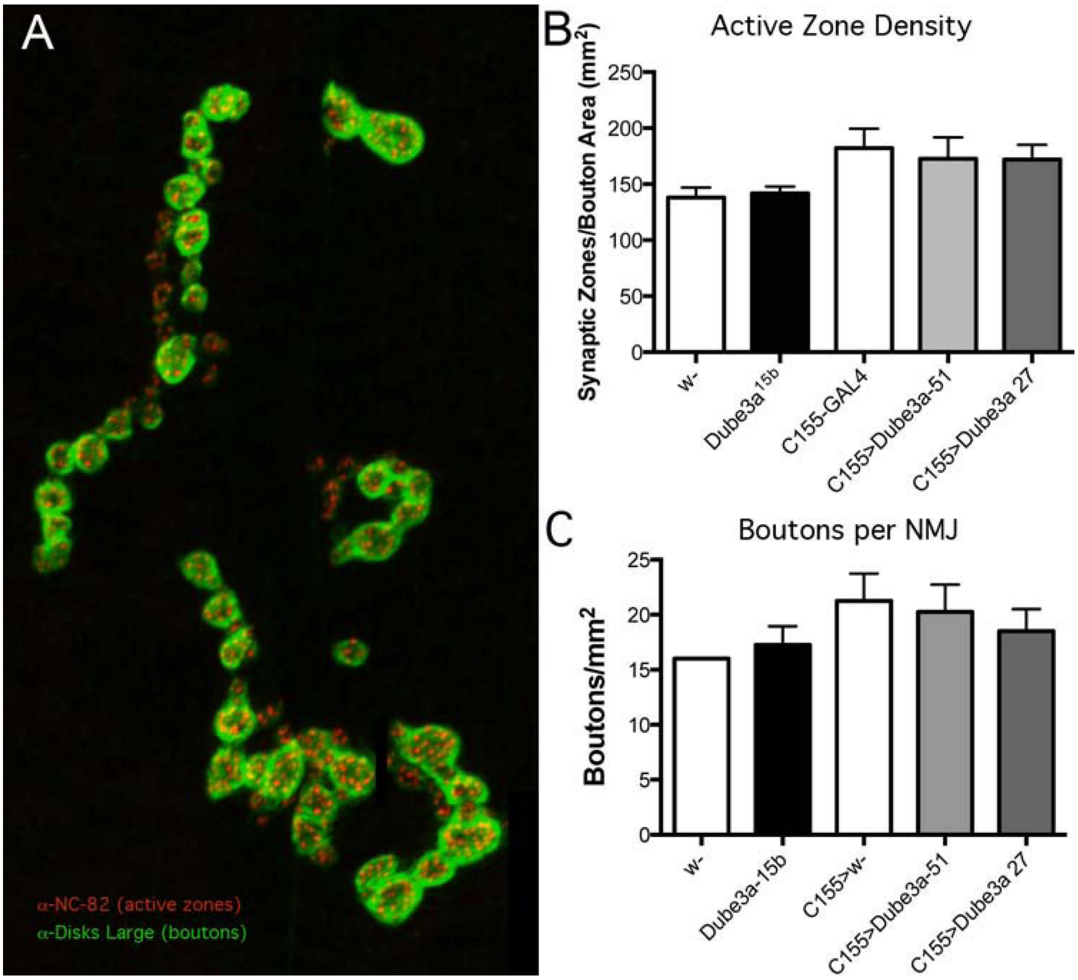

Fig. S2. Active zones per bouton and number of boutons are not affected by changes in Dube3a expression. (A) Merged image of both red channel (Nc-82) and green channel (Dlg) to show the location of the active zones (red) within the boutons (green) of a typical NMJ. The red channel represents α-Nc-82 staining used to identify synaptic active zones for quantification by “finding maxima” in ImageJ. Each spot was counted in ImageJ and quantified for analysis of active zone density. The green channel (α-Dlg) was used to identify and count the number of boutons. (B) Quantification of both synaptic active zone density (top) and number of boutons (bottom) in all genotypes. Error bars are standard error of the mean. No significant differences were identified among genotypes.
